# Nonlinear Time Domain Relation between Respiratory Phase and Timing of the First Heart Sound

**DOI:** 10.1155/2015/410102

**Published:** 2015-10-01

**Authors:** Hong Tang, Yongwan Park, Chengjie Ruan

**Affiliations:** ^1^Department of Biomedical Engineering, Dalian University of Technology, No. 2 Linggong Road, Dalian 116024, China; ^2^Department of Information and Communication Engineering, Yeungnam University, Dae-dong, Gyeongsan-si, Gyeongsangbuk-do 712749, Republic of Korea

## Abstract

The previous studies on respiratory physiology have indicated that inspiration and expiration have opposite effects on heart hemodynamics. The basic reason why these opposite hemodynamic changes cause regular timing variations in heart sounds is the heart sound generation mechanism that the acoustic vibration is triggered by heart hemodynamics. It is observed that the timing of the first heart sound has nonlinear relation with respiratory phase; that is, the timing delay with respect to the R-wave increases with inspiration and oppositely decreases with expiration. This paper models the nonlinear relation by a Hammerstein-Wiener model where the respiratory phase is the input and the timing is the output. The parameter estimation for the model is presented. The model is tested by the data collected from 12 healthy subjects in terms of mean square error and model fitness. The results show that the model can approximate the nonlinear relation very well. The average square error and the average fitness for all the subjects are about 0.01 and 0.94, respectively. The timing of the first heart sound related to respiratory phase can be accurately predicted by the model. The model has potential applications in fast and easy monitoring of respiration and heart hemodynamics induced by respiration.

## 1. Introduction

Heart sounds are commonly considered as a series of mechanical vibrations produced by heart vascular system [1–4]. The vibrations may be generated by the rapid contraction and extension of heart wall, blood turbulence in heart chamber and great vessel, and valves' vibration triggered by blood pressure difference. In this reasoning, a change in heart hemodynamics is possible to be reflected by the features (amplitude, timing, split, frequency, etc.) of heart sounds. Heart hemodynamic changes can be caused by physical activities (running, walking, etc.) [5], medicine (epinephrine, etc.) [2, 3, 6], and physiological activities (respiratory) [7, 8]. A normal respiratory process generally causes regular heart hemodynamic changes. During inspiration, an increased pressure gradient is observed from the extrathoracic regions to the right atrium because of the lowered pleural pressure. This increased gradient leads to increased blood filling of the right ventricle (RV). The increased RV end-diastolic volume (EDV) leads to an increased RV stroke volume (SV) via the Frank-Starling mechanism. The dilated RV causes the left ventricle (LV) to become less compliant by physical compression. The interventricular septum thus moves leftward, which results in reduced LV filling. Simultaneously, the distending lungs and their circulatory volume tend to reduce the pressure gradient and flow from the pulmonary veins to the LV, and the transmural diastolic aortic pressure, which is the LV afterload, increases. These additive effects induced by respiration result in decreased LV-SV. The opposing process occurs during expiration in which RV-EDV and RV-SV decrease and LV-EDV and LV-SV increase. Due to these regular hemodynamic changes caused by respiratory process, features of heart sounds have close relation to the respiratory process. The relation is especially prominent as a body in peace and quiet conditions because of less interference. The mechanism of the splitting of S2 induced by respiration was well established in [9]. Greater splitting of S2 was observed during inspiration due to the earlier occurrence of the aortic component and a delay in the pulmonary component. Respiration has also been shown to modulate systolic and diastolic time intervals [7, 8]. Amit et al. [8] characterized the morphological variations induced by respiratory activity using the computational techniques of cluster analysis and classification. The authors' study further proved that respiration has regular effect on the timing of the first heart sound (S1) [7]. The delay of S1 reached a maximum at late inspiration and reached a minimum at late expiration and the aortic component occurred earlier in inspiration and later in expiration. The delays of the first heart sound gradually increased with inspiration and reached a maximum at the end of inspiration; the opposite behavior was observed in expiration. The delay at deepest inspiration was significantly greater than that at peak expiration with all subjects who were involved.

It can be found from the previous works that the relation between respiratory phase and the timing of S1 was highly nonlinear. This paper tries to approximate the relation in time domain by quantitative nonlinear Hammerstein-Wiener model. The timing of the first heart sound may therefore be predicted by the model. Cardiovascular status in respiratory process may be possible to be monitored by the relation. These results suggest that a quantitative analysis of the relation could be used as a noninvasive continuous monitoring of hemodynamic state during respiratory cycles.

## 2. Methods

### 2.1. Data Collection

**Figure 1:**
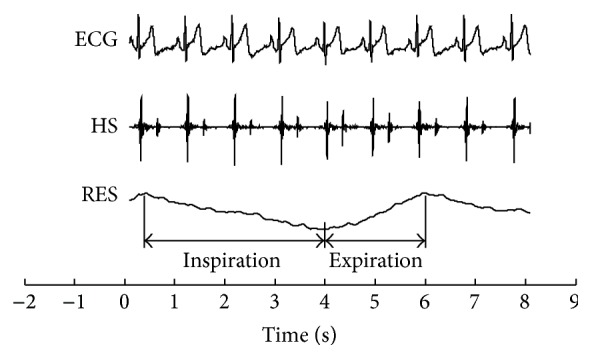
A portion of the collected signals. ECG: ECG signal of the lead II; HS: heart sound signal; RES: respiratory signal.

The experimental protocol was approved by the Ethics Committee of the Department of Biomedical Engineering, Dalian University of Technology. Twelve young male subjects aged 24 ± 1.8 years participated in the experiments. All subjects provided their consent to participate in the experiments. They were asked to remain at rest for 10 min before data collection. Each subject was asked to lie on his back in a bed during data sampling. Heart sounds, ECG lead II, and respiratory signals were simultaneously recorded at a sampling frequency of 2 kHz (PL3516B111, ADinstruments, Australia). A heart sound microphone sensor (MLT201, ADinstruments, Australia) was placed at the left third intercostal space. The breathing transducer (MLT1132, ADinstruments, Australia) was a belt sensor positioned at the middle of the thorax to record respiratory movement. One data collection period lasted 150–180 s. Data were collected thrice for each subject with 3-minute intervals. A portion of collected signals is given in Figure 1.

### 2.2. Preprocessing and Variable Definitions

**Figure 2:**
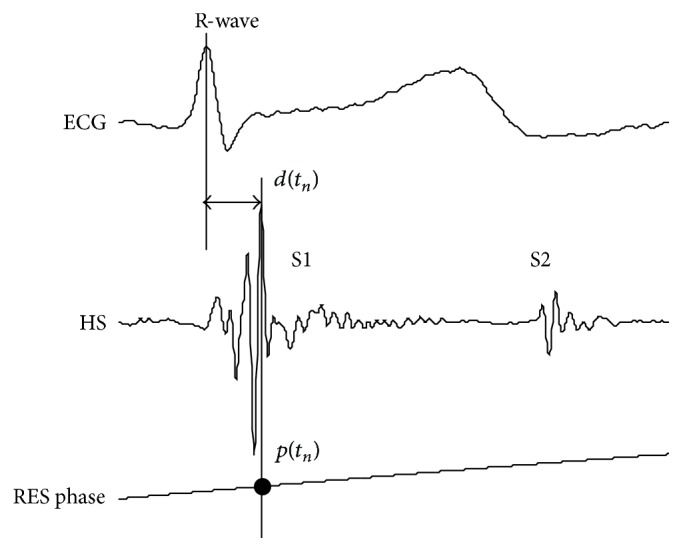
Definitions of the timing and the phase.

**Figure 3:**
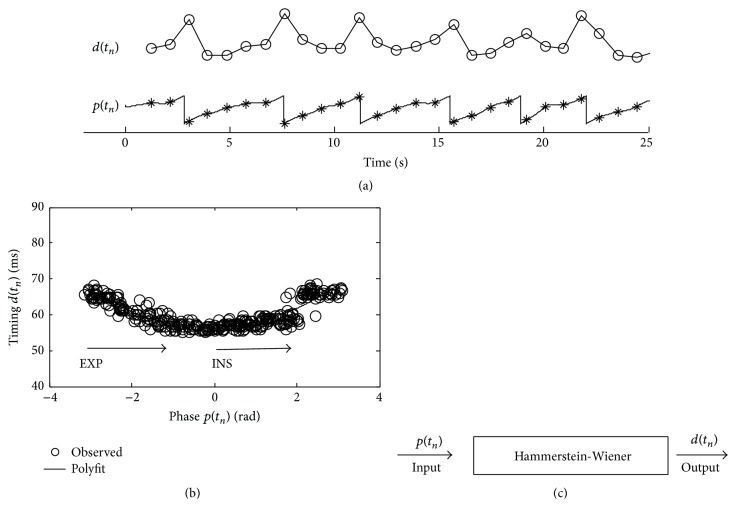
Nonlinear relation between the timing and the phase. (a) Two series of the timings and the phases are shown in time domain. (b) Scatter plot in the joint plane of timing and phase. EXP: expiration, INS: inspiration. (c) The nonlinear relation is approximated by a Hammerstein-Wiener model.

(*1) Respiratory Phase.* The respiration signal was collected from the belt sensor. The signal is band-pass filtered at [0  0.5] Hz and then subjected to a Hilbert transform. The instantaneous respiratory phase can be obtained using a 4-quadrant inverse tangent. A breathing signal was mapped to the respiration phase [−*π* *π*]. This study defines the respiration phase: inspiration begins at phase 0 and ends at phase *π* (maximum negative intrathoracic pressure), and expiration begins at phase −*π* and ends at phase 0 (maximum positive intrathoracic pressure).

(*2) Variables' Definitions.* The timing of the first heart sound is defined as the time delay from the R-wave to the prominent peak of the first heart sound. It is denoted as *d*(*t*_*n*_) in Figure 2, where the variable “*t*_*n*_” is the time occurrence at which the prominent peak of the first heart sound of the *n*th cardiac cycle occurs. The respiratory phase corresponding to the prominent peak is denoted as *p*(*t*_*n*_), shown in Figure 2. A pair of *d*(*t*_*n*_) and *p*(*t*_*n*_) can be extracted from one cardiac cycle. Discrete time series of *d*(*t*_*n*_) and *p*(*t*_*n*_) can thus be obtained from the data recordings (seen in Figure 3(a)).

### 2.3. Hammerstein-Wiener Model

**Figure 4:**
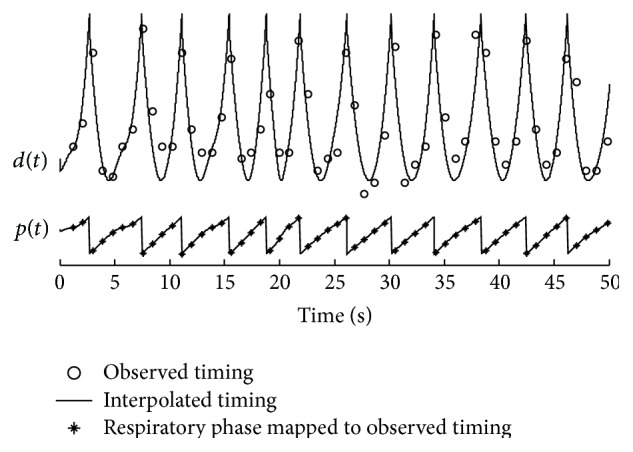
Sampling frequency transform from nonuniform discrete to uniform discrete by interpolation.

(*1) Nonlinear Relation between d*(*t*_*n*_)* and p*(*t*_*n*_). To view the nonlinear relation between *d*(*t*_*n*_) and *p*(*t*_*n*_), an example is given in Figure 3(b). Over three hundred pairs of *d*(*t*_*n*_) and *p*(*t*_*n*_) were extracted from signals collected from a subject. The scatter plot of the data was drawn in the joint phase-timing plane as shown in Figure 3(b). It can be seen that the timing varies with the respiratory phase. This is originated from the regular hemodynamic changing in heart chambers and great vessel induced by respiration. This phenomenon was observed by the authors in all the subjects involved in the experiments [7]. To view the trend of respiratory phase with respect to the associated timing, a polynomial fitting is used to approximate the relation as indicated by the solid line in Figure 3(b). The timing delay gradually increased with inspiration and reached a maximum at the end of inspiration; the opposite behavior was observed in expiration. The purpose of this paper is to study the time domain relation between *d*(*t*_*n*_) and *p*(*t*_*n*_) quantitatively. The timing, *d*(*t*_*n*_), was expected to be predicted by *p*(*t*_*n*_) via a nonlinear Hammerstein-Wiener model (seen in Figure 3(c)). 

(*2) Sampling Frequency Transform.* One pair of data, *d*(*t*_*n*_) and *p*(*t*_*n*_), can be obtained from one cardiac cycle. A problem rises that the discrete sequences of *d*(*t*_*n*_) and *p*(*t*_*n*_) are not uniformly discrete in time domain because the time interval of the digital sequence *d*(*t*_*n*_) is not a fixed number. The reasons to explain this nonuniform discrete are (1) the peak timings with respect to the R-waves are varying from one cardiac cycle to another and (2) the cardiac durations are varying from cycle to cycle due to the heart rate variability. To overcome this problem, a sampling frequency transform is needed. From the scatter plot shown in Figure 3(b), one can see that the relation looks symmetric with respect to phase zero. A second-order polynomial is thus good to fit the relation. The uniformly discrete data can be obtained by the polynomial interpolation. On the other hand, the fitting may be considered as a way to reduce the errors caused by unknown reasons. Once the sampling frequency transform is completed, the discrete sequence of respiratory phase and the timing sequence are uniformly discrete in time domain, seen in Figure 4. They can be used as the input and output of the model. The two discrete sequences after sampling frequency transform are denoted as *p*(*t*) and *d*(*t*) in the following.

**Figure 5:**
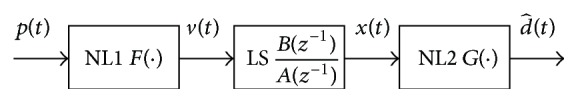
Structure of the Hammerstein-Wiener model.

A Hammerstein-Wiener model generally consists of three parts [10, 11], that is, a static nonlinear module (NL1), a dynamic linear module (LS), and another nonlinear module (NL2), seen in Figure 5. With the three modules, this model structure has the ability to approximate a high dynamic nonlinearity [11]. The phase sequence *p*(*t*) is input to the first nonlinear module NL1 and the output is *v*(*t*). The internal *v*(*t*) is the input of dynamic linear module LS which outputs *x*(*t*). The nonlinear module NL2 has input *x*(*t*) and output *d*(*t*). In the model, *v*(*t*) and *x*(*t*) are internal variables. The variable transmissions between the modules are written as

(1) vt=Fpt,

(2) xt=Bz−1vt+1−Az−1xt,

where

(3) Az−1=1+a1z−1+⋯+amz−m,Bz−1=b0+b1z−1+⋯+bnz−n.

The predicted timing is the output of the model

(4) $$\begin{array}{c} \hat{d}\left(\;t\;\right)=G\left(\;x\left(\;t\;\right)\;\right). \end{array}$$

(*3) Parameter Estimation of the Model.* In order to achieve high nonlinearity, the nonlinear modules NL1 and NL2 are assumed to be polynomials with known order *N*_*F*_ and *N*_*G*_. It is obvious that the degree of nonlinearity that the Hammerstein-Wiener model can reach is determined by the orders of the polynomials. Generally speaking, the higher degree of nonlinearity between the input and the output and the higher orders are needed:

(5) vtFpt=∑i=1NFfipit,

(6) d^tGxt=∑i=1NGgixit.

The output of the linear module is rewritten as

(7) xt=b0vt+Bz−1−b0vt+1−Az−1xt.

Equation (1) is submitted into (7); one can obtain

(8) xt=b0Fpt+Bz−1−b0vt+1−Az−1xt.

The nonlinear module *G*(·) is rewritten as

(9) $$\begin{array}{c} \hat{d}\left(\;t\;\right)=g_{1}x\left(\;t\;\right)+\overset{N_{G}}{\underset{i=2}{\sum }}g_{i}x^{i}\left(\;t\;\right). \end{array}$$

Equation (7) is submitted into (9); one can obtain
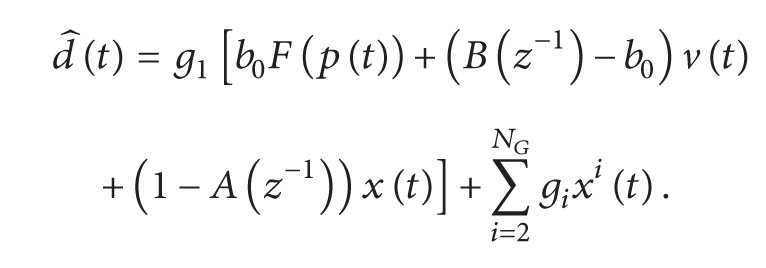
(10)To simplify the relation shown in (10), it commonly assumes that the variable *g*_1_ = 1 and *b*_0_ = 1. Equation (5) is submitted into (10); the output of the model is finally presented as

(11) d^t=∑i=1NFfipit+Bz−1−1vt+1−Az−1xt+∑i=2NGgixit,

where $\hat{d}(t)$ is the predicted timing by the model. The predicted timing can be organized as a product of vectors

(12) $$\begin{array}{c} \hat{d}\left(\;t\;\right)=\varphi^{T}\left(\;t,\theta \;\right)\theta , \end{array}$$

where ***θ*** is a parameter vector

(13) $$\begin{array}{c} \theta ={\left[\;f_{1},\ldots ,f_{N_{F}},b_{1},\ldots ,b_{n},a_{1},\ldots ,a_{m},g_{2},\ldots ,g_{N_{G}}\;\right]}^{T}. \end{array}$$

The operator “T” is matrix transposition. *φ*(*t*, ***θ***) is the data vector

(14) φt,θ=pt,…,pNFt,vt−1,…,vt−n,−xt−1,…,−xt−m,x2t,…,xNGtT.

There are two internal variables which are generally unmeasurable. Hence, the parameter estimation cannot be performed directly on the basis of  (12). The previous work applies an iterative approach with internal variable estimation to estimate the parameters [11]. This approach can be simply extended to the case of two or even more internal variables.

The iterative approach is implemented based on the preceding estimation for internal variables. It is assumed that the internal variables of the *s*th step in the iterative procedure are

(15) vts=∑i=1NFfispit,xst=vst+∑i=1nbisvst−i−∑j=1majsxst−j.

The error to be minimized in the *s*th step is written as

(16) ets=dt−φTst,θsθs,

where ^*s*^ *φ*(*t*, ^*s*^ ***θ***) is the data vector with the corresponding estimates of internal variables according to (15).

The steps to implement the iterative procedure may be summarized as follows.

(1) Initialization: the parameter *θ* is initialized as zero vector and *s* = 1.
(2) A least mean square (LMS) algorithm is used to minimize the error in the *s*th step.
  (a) Starting from *t* = *m* + *n* + *N*_*F*_ + *N*_*G*_ − 1, let ^*s*^ ***θ***(*t*) = ^*s*^ ***θ***.
  (b) An iterative procedure is used to obtain the optimal parameter for the *s*th step
    (17) est=dt−φTst,θstθst,θst+1=θst+μestφst,θst,
   
*μ* is a step size in the procedure.
  (c) Once the iteration converges or reaches the maximum number of iterations, the iteration stops. The convergence condition is generally defined as the degree of variation of the parameters in the process, such as the dynamic variance. Once the degree of variation reaches a low level, the iteration is believed to converge successfully. The optimal parameter vector, ^*s*^ ***θ***_opt_, for the *s*th step is obtained.
(3) Let ^*s* + 1^ ***θ*** = ^*s*^ ***θ***_opt_. ^*s* + 1^ ***θ*** is substituted into (15) to obtain the internal variable ^*s* + 1^ **v**(*t*) and ^*s* + 1^ **x**(*t*).
(4) Repeat the steps (2)–(4).

### 2.4. Performance Evaluation

In this model, respiratory phase is the model input and the timing is the model output. Two indicators are used to evaluate the consistence between the predicted timing and the observed timing. One indicator is mean square error (MSE) which is written as

(18) $$\begin{array}{c} \text{MSE}=\frac{1}{N}\overset{N}{\underset{t=1}{\sum }}{\left(\;d\left(\;t\;\right)-\hat{d}\left(\;t\;\right)\;\right)}^{2}, \end{array}$$

where *N* is number of samples of the digital sequence. Another indicator is the degree of fitness which is

(19) $$\begin{array}{c} R^{2}=1-\frac{\sum_{t=1}^{N}{\left(d\left(t\right)-\hat{d}\left(t\right)\right)}^{2}}{\sum_{t=1}^{N}{\left(d\left(t\right)-\overset{-}{d}\right)}^{2}}, \end{array}$$

where $\overset{-}{d}$ is the average of *d*(*t*). The degree of fitness is in the range [0  1]. A higher fitness value means that the model can approximate the relation between the input and output better.

## 3. Experiments and Discussions

### 3.1. Experimental Results

**Figure 6:**
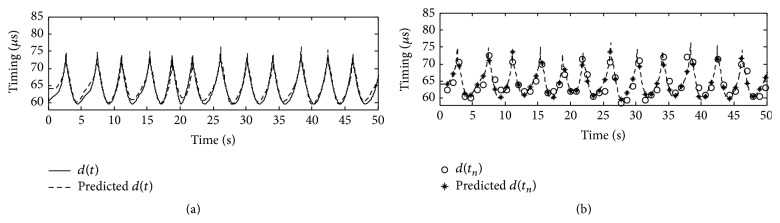
An example of model evaluation. (a) Approximation between *d*(*t*) and the predicted *d*(*t*), (b) approximation between the observed timing and the predicted timing.

The digital sequence of respiratory phase and timing are both slow varying. They are downsampled to 50 Hz to reduce the number of samples. The time domain nonlinear relation between respiratory phase and timing of S1 is virtually seen in Figure 3(a). The authors analyzed the nonlinear relations for various subjects. It was found that a five-order polynomial was sufficient to approximate the nonlinearity and even the nonlinearity varied from one subject to another. Based on this preknowledge, the polynomial order in the nonlinear blocks is set to 5; that is, *N*_*F*_ = 5 and *N*_*G*_ = 5. In a similar way, the delay orders in the linear block can also be analyzed by the relative delay in time domain between the input and output. The delay orders are empirically set as *m* = 10 and *n* = 40. The data was collected from a healthy male subject aged 23 years. The respiratory phase sequence, *p*(*t*), was input to the model with the abovementioned settings. The interpolated timing by the solid line and predicted timing indicated by dash line are shown in Figure 6(a). The predicted timing indicated by “*∗*” and observed timing indicated by “O” are shown in Figure 6(b). It can be seen that the stars are close to the corresponding circles. The MSE and fitness measured for Figure 6 are 0.01 and 0.94, respectively. It means that the model works very well to approximate the high dynamic nonlinearity for the relation between respiratory phase and S1 timing.

There are 12 subjects involved in the experiments to collect data. To evaluate the model adaption to other subjects, the MSE and fitness were listed in Table 1.

### 3.2. Discussions

**Figure 7:**
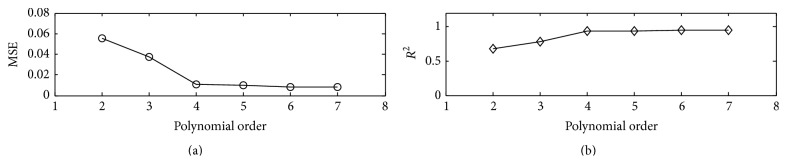
Approximation performance of the Hammerstein-Wiener model for subject #2 with varied polynomial order. (a) MSE decreased with the polynomial order; (b) fitness value increased with the polynomial order.

The degree of nonlinearity that the Hammerstein-Wiener model can approximate is directly related to the order of the polynomials in the first and third modules. The basic understanding to the model shows that a higher degree of nonlinearity needs higher order polynomials. However, the degree of nonlinearity between the respiratory phase and the timing of S1 varies from person to person. Then the question of how the order of the polynomials can be selected arises. The authors analyzed the nonlinearity that the Hammerstein-Wiener model approximates for all subjects. The results showed that the approximation performance of the model increased a little once the order of the polynomials is greater than 3. For example, for subject #2 in Table 1, the variations of the performance are shown in Figure 7 when the order of polynomials increases from 2 to 7. It can be found that significant improvement was obtained as the order increases from 2 to 4; however, the improvement is much less and even cannot be detected by visual check as the order increases from 4 to 7. The similar performance was observed for the other 11 subjects. So, the authors conclude that the order selection is not a problem. Excellent performance is expected to be obtained if the order is greater than 4.

## 4. Conclusions

The studies in respiratory physiology indicated that respiration has regular effect on heart hemodynamics. These hemodynamic variations lead to the fact that the closure of mitral valve occurs late in inspiration and early in expiration. The opposite behaviors of mitral valve close are further reflected by the timing of first heart sound due to the mechanism of heart sound generation. A nonlinear relation is observed between the timing and the respiratory phase based on data collected from healthy subjects. A Hammerstein-Wiener model is used to approximate the nonlinear relation in time domain. The parameter estimation for the model is presented in this paper. The performance tests show that the timing of first heart sound can be accurately predicted by the respiratory phase based on the model. This has potential applications in fast and easy monitoring of respiration and heart hemodynamics induced by respiration.

## Figures and Tables

**Table 1:** MSE and fitness between *d*(*t*) and predicted *d*(*t*) for 12 subjects.

Subject number	1	2	3	4	5	6	Average
MSE	0.008	0.009	0.011	0.006	0.014	0.006	0.009
*R* ^2^	0.963	0.957	0.946	0.970	0.926	0.959	0.954

Subject number	7	8	9	10	11	12	Average

MSE	0.019	0.016	0.010	0.011	0.014	0.010	0.013
*R* ^2^	0.921	0.925	0.952	0.945	0.934	0.949	0.938
